# Hemoporfin-mediated photodynamic therapy with general anesthesia showed superior efficacy in the treatment of port-wine stains: a retrospective evaluation

**DOI:** 10.3389/fmed.2023.1170520

**Published:** 2023-05-24

**Authors:** Yan-Yan Hu, Kai Chen, Lin-Lin Wang, Jia-Fang Wang, Xi Chen, Li-Juan Cao, Qian Jiang, Zhen-Xing Wang, Shan-Shan Qian, Zhi-Jun Chen, Liu-Qing Chen, Dong-Sheng Li

**Affiliations:** ^1^Department of Dermatology, Wuhan No. 1 Hospital, Tongji Medical College, Huazhong University of Science and Technology, Wuhan, China; ^2^Hubei Key Laboratory of Infectious and Immune Skin Diseases, Wuhan No. 1 Hospital, Tongji Medical College, Huazhong University of Science and Technology, Wuhan, China; ^3^Department of Anesthesiology, Wuhan No. 1 Hospital, Tongji Medical College, Huazhong University of Science and Technology, Wuhan, China; ^4^Department of Anesthesiology, Tongji Hospital, Tongji Medical College, Huazhong University of Science and Technology, Wuhan, China; ^5^School of Medicine, JiangHan University, Wuhan, China

**Keywords:** photodynamic therapy, hemoporfin, general anesthesia, port-wine stains, efficacy

## Abstract

**Background:**

Hemoporfin-mediated photodynamic therapy (PDT) is an effective treatment for port-wine stains (PWS), and pain is the main adverse effect of this therapy. General anesthesia is commonly used for pain management during PDT, but the effect of general anesthetics on the subsequent treatment efficacy of PDT in PWS has not been reported.

**Objectives:**

To assess the use of general anesthesia combined with PDT compared with PDT alone in 207 PWS patients, and to provide further safety and efficacy data on this combined therapy.

**Methods:**

Propensity score matching (PSM) was used at a 2:1 ratio to create a general anesthetic group (*n* = 138) and a highly comparable nonanesthetic group (*n* = 69). The clinical outcomes were evaluated, and the treatment reactions and adverse effects were recorded after one treatment with PDT.

**Results:**

After matching, there was no significant difference in the demographic data of the patients in the two groups (*p* > 0.05), while the treatment efficacy was significantly higher in the general anesthetic group than in the nonanesthetic group (76.81 vs. 56.52%, *p* < 0.05). Moreover, logistic regression analysis confirmed that patients receiving general anesthesia showed an association with a good response to PDT (OR = 3.06; 95% CI, 1.57–6.00; *p* = 0.0011). Purpura lasted longer in the general anesthetic group, but the other treatment reactions and adverse effects were similar in the two groups (*p* > 0.05). No serious systemic adverse reactions were observed.

**Conclusion:**

We recommend this combined therapy, which is associated with painless, as a high efficacy treatment option for PWS patients, especially for patients with a poor response to multiple PDT alone treatments.

## 1. Introduction

Port-wine stains (PWS) are a common congenital capillary malformation that often occur on the face and neck, and they have an incidence of 3–5/1,000 in neonates globally (1). Although pulsed dye laser (PDL) has been considered as the gold standard treatment for PWS, only 10–20% of skin lesions achieve a complete clearance after repeated rounds (2, 3). With better clinical outcomes and fewer treatment sessions, hemoporfin-mediated photodynamic therapy (HMME-PDT) has now been a novel effective treatment alternative for PWS birthmarks in China (4, 5). However, during the course of PDT, most patients experience burning and tingling pain that occurs approximately 10 min after light irradiation, and this pain rapidly exacerbates to a peak and lasts until the end of treatment (6). In some cases, patients frequently move or even interrupt treatment because of the unbearable pain, and this not only affects the treatment efficacy but also causes a huge mental burden to patients and their families (7). Therefore, effective pain management strategies during PDT are required.

Pain during PDT is the main limiting side-effect in its use in dermatology. Several strategies for controlling the pain during treatment have been explored, such as cold-air analgesia, oral or intravenously administered analgesia, and nerve blocks (8). Although some of them achieve a reduction in the levels of pain, none were completely effective and convenient (9–11). General anesthesia, the most common pain management strategy in anesthesia care, has been extensively used in clinical practice, such as for total knee replacement, lumbar laminectomy with instrumentation, exploratory laparotomy, and cesarean delivery (12). Moreover, general anesthesia combined with PDT results in pain-free treatment, but the effect of general anesthetics on the subsequent treatment efficacy of PDT has rarely been reported.

This retrospective study was conducted to evaluate the safety and efficacy of general anesthesia combined with PDT in the treatment of PWS, to provide safety guidance for clinical practice and to explore the therapeutic method for patients who have a poor response after multiple PDT alone treatments.

## 2. Materials and methods

### 2.1. Study design

Consecutive patients with PWS who received HMME-PDT treatment in the Department of Dermatology, Wuhan No. 1 Hospital, from April 2018 to May 2021 were enrolled in this retrospective study. We used a propensity score matching (PSM) method (match tolerance = 0.03) at a 2:1 ratio to create a general anesthetic group (*n* = 138) and a highly comparable nonanesthetic group (*n* = 69) based on the patient age, history of prior treatment, and the color and localization of PWS that may affect the treatment efficacy of PDT (13). This study was approved by the ethics committee of Wuhan No. 1 Hospital, Tongji Medical College, Huazhong University of Science and Technology (ChiCTR2100047966).

### 2.2. Images taken

Before and 8–12 weeks after one PDT treatment, images of the skin lesions of each patient were taken using the supplied Canon 650D SLR camera. Dermoscopic images were taken using the ATBM FotoFinder Bodystudio dermatoscope (Germany, polarized, ×20).

### 2.3. Efficacy evaluation

The treatment efficacy was assessed by using the patient images before and after one PDT treatment. The clearance of the PWS lesion was evaluated by three dermatologists in a blinded and independent manner. The degree of improvement in the treated area was evaluated according to the following four levels: (i) cured (≥90%), (ii) good effect (60–89%), (iii) alleviation (20–59%), and (iv) no effect (<20%) (14). The treatment efficacy was calculated by the sum of the cured, good effect and alleviation patients divided by the total number of patients.

### 2.4. Safety analysis

After the PDT treatment, the local or systemic adverse events were recorded in detail as previously reported (15). Local edema, purpura, blister, scab and the duration (in days) of these symptoms at the treatment site were recorded as the treatment reactions. Adverse events, such as infection, dermatitis, erythema, texture change, scar formation, hyperpigmentation, and hypopigmentation, were also recorded to monitor the adverse effects.

### 2.5. Statistical analysis

The Kruskal-Wallis test was performed to analyze the difference between the two groups, and the Wilcoxon test was used to compare the treatment efficacy on the PWS lesions after one PDT treatment. A logistic regression analysis was used to assess the independent factors for the clinical response to PDT. Data analysis was carried out using statistical analysis system (SAS) software. The data are expressed as the mean ± SD, and *p* < 0.05 was considered to indicate statistical significance.

## 3. Results

### 3.1. Patient demographic data

A total of 138 patients (61 males) with a mean age of 9.00 ± 10.30 years and 69 patients (29 males) with a mean age of 10.94 ± 11.81 years were included in the general anesthetic group and the nonanesthetic group, respectively. There were 39 cases with pink, 71 cases with red, 14 cases with purple, and 14 cases with hypertrophic-type PWS in the general anesthetic group, and the color of PWS in the nonanesthetic group were involved in 20 patients had pink, 36 had red, five had purple, and eight had hypertrophic-type PWS. Among them, 57.97% of patients had received other treatments prior to PDT. Overall, there was no significant difference in the demographic data of patients in the two groups (*p* > 0.05). The patient data and clinical characteristics are provided in Table 1.

**Table 1:** Demographic date of the 207 PWS patients in this study.

Characteristic	Nonanesthetic group (*n* = 69)	General anesthetic group (*n* = 138)	*p* value
Male, *n* (%)	29 (42.03)	61 (44.20)	0.7661
Age, years (mean ± SD)	10.94 ± 11.81	9.00 ± 10.30	0.2253
Age group (years, *n* [%])			0.8037
1–3	30 (43.48)	59 (42.75)	
4–6	11 (15.94)	29 (21.01)	
7–12	8 (11.59)	19 (13.77)	
13–18	1 (1.45)	2 (1.45)	
≥19	19 (27.54)	29 (21.01)	
Color of PWS, *n* (%)			0.9133
Pink	20 (28.99)	39 (28.26)	
Red	36 (52.17)	71 (51.45)	
Purple	5 (7.25)	14 (10.14)	
Hypertrophic	8 (11.59)	14 (10.14)	
Localization, *n* (%)			0.5079
Central face	54 (78.26)	102 (73.91)	
Lateral face	5 (7.25)	14 (10.14)	
Neck	1 (1.45)	5 (3.62)	
Limbs	8 (11.59)	17 (12.32)	
Trunk	1 (1.45)	0 (0.00)	
Previous treatment, *n* (%)	40 (57.97)	80 (57.97)	1.0000

### 3.2. Treatment efficacy and clinical features

After one treatment with PDT, 5.80% of patients were cured, 18.84% of patients showed a good effect, and 31.88% of patients showed alleviation in the nonanesthetic group. In the general anesthetic group, the number of patients who showed cure, good effects and alleviation increased to 8.70, 21.74, and 46.38%, respectively. Moreover, we found a significant increase in the treatment efficacy (cured, good effect, and alleviation) in patients under general anesthesia compared with the nonanesthetic patients (76.81 vs. 56.52%, *p* < 0.05). The logistic regression analysis also confirmed the good response of patients to general anesthesia (OR = 3.06; 95% CI, 1.57–6.00; *p* = 0.0011). These results, which are summarized in Table 2, indicated a superior treatment efficacy of general anesthesia combined with PDT in PWS patients.

**Table 2:** Efficacy outcome of HMME-PDT in the treatment of PWS in the two groups.

Photodynamic efficacy	Nonanesthetic group (*n* = 69)	General anesthetic group (*n* = 138)	*p* value
Treatment efficacy (%)			0.0252
Cured (≥90%)	4 (5.80)	12 (8.70)	
Good effect (60–89%)	13 (18.84)	30 (21.74)	
Alleviation (20–59%)	22 (31.88)	64 (46.38)	
No effect (<20%)	30 (43.48)	32 (23.19)	
Logistic analysis			0.0011
Odds ratio (OR)	1.00	3.06	
95% CI		1.57–6.00	

Confidence interval (CI).

**Figure 1:**
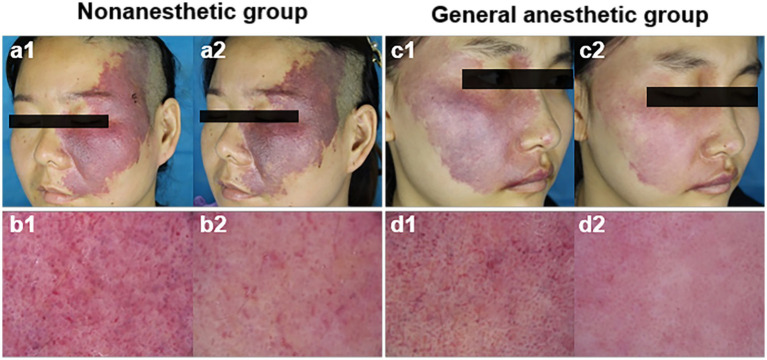
Representative dermoscopy and clinical pictures of the PWS lesions in the two groups before and after HMME-PDT. Before (a1) and after (a2) one HMME-PDT treatment, representative clinical pictures of a patient from the nonanesthetic group, and c1 and c2 represent a patient from the general anesthetic group; before (b1) and after (b2) one HMME-PDT treatment, representative dermoscopy of PWS lesions in a patient from the nonanesthetic group, and d1 and d2 represent a patient from the general anesthetic group.

Photodynamic therapy achieved great treatment efficiency in PWS patients, but the different groups of patients had markedly different responses. The lesions of the patients in the general anesthetic group were remarkably cleared, while those in the nonanesthetic group had a poor response. Figure 1 shows representative dermoscopy and clinical pictures of the PWS lesions in the two groups before and after PDT.

### 3.3. Treatment responses and adverse events

The treatment reactions and adverse events were also investigated. Of the 207 patients, 199 developed purpura, and there was no significant difference between the two groups (*p* > 0.05). However, the duration of purpura was significantly increased in the general anesthetic group compared with the nonanesthetic group (7.14 ± 3.92 vs. 5.42 ± 2.90, *p* < 0.01). These findings, as presented in Table 3, suggest a greater depth of vascular damage and better therapeutic efficacy of patients under general anesthesia. In addition, no significant differences were seen for other treatment reactions in the two groups.

**Table 3:** Treatment reactions and adverse effects between the two groups.

Evaluation indicators	Nonanesthetic group (*n* = 69)	General anesthetic group (*n* = 138)	*p* value
Treatment reactions, *n* (%)			
Edema	69 (100.00)	133 (96.38)	0.1095
Edema days (mean ± SD)	4.83 ± 2.01	4.60 ± 2.48	0.5143
Purpura	65 (94.20)	134 (97.10)	0.3078
Purpura days (mean ± SD)	5.42 ± 2.90	7.14 ± 3.92	0.0015
Blister	4 (5.80)	7 (5.07)	0.8266
Blister days (mean ± SD)	0.29 ± 1.28	0.43 ± 2.16	0.6264
Scab	29 (42.03)	66 (47.83)	0.4301
Scab days (mean ± SD)	5.01 ± 7.51	4.46 ± 6.30	0.5744
Adverse events, *n* (%)			
Infection	6 (8.70)	15 (10.87)	0.6253
Dermatitis	3 (4.35)	8 (5.80)	0.6612
Erythema	4 (5.80)	8 (5.80)	1.0000
Texture change	6 (8.70)	26 (18.84)	0.0570
Scar formation	6 (8.70)	19 (13.77)	0.2911
Hyperpigmentation	39 (56.52)	61 (44.20)	0.0945
Hypopigmentation	1 (1.45)	3 (2.17)	0.7211

All patients developed various kinds of adverse events after PDT, including infection, dermatitis, erythema, texture change, scar formation, hyperpigmentation, and hypopigmentation. No other systemic adverse reactions were observed. Importantly, except for a slight increase in the incidence of skin textural changes in the general anesthetic group (18.84 vs. 8.70%, *p > 0.05*), the occurrence rates of the other adverse events were similar in the two groups (Table 3). Taken together, our clinical data demonstrated that general anesthesia combined with PDT is a safe and effective treatment option for PWS patients.

## 4. Discussion

This retrospective analysis of 207 PWS patients treated with HMME-PDT was the first study concerning the therapeutic efficacy of PDT with general anesthesia. Our results show that general anesthesia combined with PDT results in a safe and effective outcome. There was a significant increase in the treatment efficacy in patients under general anesthesia, and no serious adverse reactions were observed. Although the Food and Drug Administration (FDA) has warned about the risks of general anesthesia to the neurodevelopmental outcomes of young children (16), we did not observe any obvious neuropsychological and behavioral deficits after the treatment. Consistent with our data, an international, randomized controlled trial previously provided strong evidence that transient exposure to general anesthesia in infancy does not alter the neurodevelopmental outcomes (17). Moreover, another Mayo Anesthesia Safety in Kids (MASK) study found that general anesthesia exposure prior to the age of 3 years was not associated with behavioral and learning difficulties (18). In our treatment, the patients were exposed to general anesthesia for less than 1 h and received no more than five anesthetic events per year. Thus, we believe that general anesthesia combined with PDT is a safe therapy for PWS patients.

Photodynamic therapy is a modern and noninvasive technology that combines a photosensitizer with light to generate abundant reactive oxygen species (ROS) to clear damaged tissues (19). In this study, we used different oxygen inhalation apparatuses, such as endotracheal tube, face mask, laryngeal mask airway and high-flow nasal cannula, to maintain a high oxygen partial pressure in the patients under general anesthesia. We found that the patients in the general anesthetic group had a significantly increased treatment efficacy compared with those in the nonanesthetic group, but the underlying mechanism remains unclear. Previous studies reported that the influential factors of treatment efficacy include the clinician’s treatment parameters, patient’s cooperation and the oxygen concentrations. Moreover, Maier and his colleagues found that hyperbaric oxygen (HBO) enhances the photodynamic effect of PDT in patients with advanced esophageal carcinoma (20). In another experimental animal study, Jingqiu et al. demonstrated that synergizing upconversion nanophotosensitizers (UNPSs) with HBO improved the efficacy of photodynamic cancer therapy by facilitating the diffusion of oxygen and the penetration depth of UNPSs (21). On the other hand, HBO combined with PDT inhibited human squamous cell proliferation (22). Thus, the key to enhancing the treatment efficacy of patients in the general anesthetic group was high oxygen inhalation, which resulted in HBO in the target tissue. These data support the conclusion that general anesthesia combined with PDT showed superior efficacy in the treatment of PWS patients.

Purpura is a common treatment response to PDT that is caused by the extravasation of blood and indicates the therapeutic endpoint of treatment (23). A previous prospective study revealed that the purpuric settings had higher efficacy in the treatment of PWS than the nonpurpuric settings (24). Here, we found that the occurrence rate of purpura was similar in the two groups, but the duration of purpura were significantly increased in the general anesthetic group compared with the control group. These data suggest that there was more extensive blood vessel destruction and a greater depth of tissue cell damage, further demonstrating the good response of patients to general anesthesia. Additionally, compared to nonanesthetic patients, we also observed a slight skin textural change in the general anesthesia patients. It has been reported that skin texture features objectively represent the skin barrier function and accurately reflect the outcomes of PDT (25). Moreover, PDT treatment led to transepidermal water loss (TEWL) and affected the skin barrier function (26). In this study, although the treatment-associated textural changes were not significant, we also recommend that PWS patients use some skin protection creams to repair the skin barrier after this combined therapy.

In conclusion, our findings demonstrate that general anesthesia combined with PDT represents a promising therapeutic option for PWS patients due to its good safety and superior efficacy. This combined therapy will provide new hope for patients who have a poor response after multiple PDT alone treatments.

## Data availability statement

The original contributions presented in the study are included in the article/supplementary material, further inquiries can be directed to the corresponding authors.

## Ethics statement

The studies involving human participants were reviewed and approved by Ethics Committee of Wuhan No. 1 Hospital, Tongji Medical College, Huazhong University of Science and Technology. Written informed consent to participate in this study was provided by the participants’ legal guardian/next of kin. Written informed consent was obtained from the individual(s) and minor(s)’ legal guardian/next of kin, for the publication of any potentially identifiable images or data included in this article.

## Author contributions

L-QC and D-SL initiated and designed the study. Y-YH, L-LW, J-FW, XC, L-JC, and QJ collected sample and validated clinical data. KC, Z-XW, S-SQ, and Z-JC performed the experiments and analyzed the data. KC and D-SL wrote the manuscript and revised it. All authors contributed to the article and approved the submitted version.

## Funding

This study was supported partially by the National Natural Science Foundation of China (81573062 and 82203926), the Knowledge Innovation Project of Wuhan Science and Technology Bureau (2022020801010524), the Innovation Groups of the Natural Science Foundation of Hubei Province (2022CFA037) and the Hubei Provincial Natural Science Foundation of China (2022CFB955).

## Conflict of interest

The authors declare that the research was conducted in the absence of any commercial or financial relationships that could be construed as a potential conflict of interest.

## Publisher’s note

All claims expressed in this article are solely those of the authors and do not necessarily represent those of their affiliated organizations, or those of the publisher, the editors and the reviewers. Any product that may be evaluated in this article, or claim that may be made by its manufacturer, is not guaranteed or endorsed by the publisher.
